# Factors associated with academic burnout and its prevalence among university students: a cross-sectional study

**DOI:** 10.1186/s12909-023-04316-y

**Published:** 2023-05-06

**Authors:** Zheng Liu, Yujin Xie, Zhuhong Sun, Di Liu, Hang Yin, Lei Shi

**Affiliations:** 1 College of Computer Science and Technology, Harbin Engineering University, Harbin, China; 2 Beijing Rehabilitation Hospital, Capital Medical University, Beijing, China; 3 College of Life Science, Northeast Agricultural University, Harbin, China; 4 School of Marxism, Harbin Medical University, Harbin, China; 5 Department of Human Resources, Sixth Affiliated Hospital of Harbin Medical University, Harbin, China; 6 School of Health Management, Southern Medical University, Guangzhou, China

**Keywords:** Academic burnout, Prevalence, Factors associated, University students, China

## Abstract

**Background:**

This study aimed to evaluate the current state of academic burnout among Chinese college students and its influencing factors.

**Methods:**

A cross-sectional study of 22,983 students was conducted using structured questionnaires and the Maslach Burnout Inventory General Survey on sociodemographic characteristics, educational process, and personal aspects. Multiple variables were statistically evaluated using logistic regression analysis.

**Results:**

The total score of the students’ academic burnout was 40.73 (± 10.12) points. The scores for the reduced personal accomplishment, emotional exhaustion, and cynicism were 23.63 (± 6.55), 11.20 (± 6.05), and 5.91 (± 5.31), respectively. Students with academic burnout accounted for 59.9% (13,753/22,983). Male students had higher burnout scores than female students, upper-grade students had higher burnout scores than lower-grade students, and students who smoked had higher burnout than non-smokers during the school day.

**Conclusions:**

More than half of students experienced academic burnout. Gender, grade, monthly living expenses, smoking, parents’ education level, study and life pressures, and the current degree of professional knowledge interest significantly impacted academic burnout. An effective wellness program and an annual long-term burnout assessment may sufficiently reduce student burnout.

**Supplementary Information:**

The online version contains supplementary material available at 10.1186/s12909-023-04316-y.

## Introduction

College is a crucial stage of personal development with increasing pressure to acquire knowledge and skills. In addition to academic demands, other challenges, such as peer pressure and competition, limited socioeconomic power, and distance from home and family, act as stressors for students. Academic burnout has become an essential factor that affects college students’ mental health. “Job burnout” was first proposed by the American clinical psychologist Fredenbeger in 1974 [1]. Maslach’s three-dimensional theory was initially used to describe burnout in the professional helpers’ industry, and its core contents were individual emotional exhaustion, depersonalisation, and personal achievement reduction. Maslach (1982) defined burnout as a syndrome of exhaustion comprising emotional exhaustion, depersonalisation, and low personal accomplishment [2]. Pines (1980) and Meier (1985) defined “academic burnout” as the exhaustion of students’ energy due to long-term academic pressure and burden, the gradual loss of enthusiasm for schoolwork and activities, indifference and alienation from classmates, and lack of enthusiasm for schoolwork [3, 4]. Soffeli (2002) proposed that academic burnout combines emotional exhaustion, cynicism, and academic inefficiency due to the persistent failure to effectively manage learning stress [5]. During compulsory education, some students experience temporary stress that prompts them to lose interest and commitment and doubt their ability to meet academic requirements. Taiwan scholar Yang Huizhen defined academic burnout as students’ emotional exhaustion, lack of humanisation, and low personal sense of achievement due to academic pressure, academic load, or other personal psychological factors in the learning process [6]. The Maslach Burnout Inventory General Survey (MBI-GS) was adopted to measure academic burnout and modified the place, object, and nature of work in the scale. Moreover, she believed that the items of this scale were more suitable for students’ situations than the scale focusing on education. Based on previous studies, our study defined academic burnout as students’ emotional exhaustion, cynicism, and low personal accomplishment due to academic pressure, academic load, or other personal psychological factors in the learning process.

A study of 4,061 college students from different countries and regions found that maintaining low burnout levels could effectively prevent students from dropping out [7]. Another study indicated that the prevalence of burnout syndrome and depressive symptoms among medical students in Oman were 7.4% and 24.5%, respectively [8]. The adjusted prevalence of burnout among 872 students in 15 public schools in Sri Lanka was 28.8% [9]. A total of 187 students (70.6%) out of 330 first-year medical students in Brazil showed high levels of emotional exhaustion, 140 (52.8%) showed high levels of cynicism, and 129 (48.7%) showed low academic performance [9]. Of 468 undergraduate chemistry students in Nigeria, 245 (54.3%) met the eligibility criteria for severe burnout [10].

Receiving a university education in China is a solemn undertaking. Students must pass challenging exams to enter high school and university to determine their future career choices. Simultaneously, the quality of school teaching is often evaluated based on student grades. Therefore, schools, teachers, and students face enormous pressure. Researchers have repeatedly found that academic burnout has become a common problem among college students in their educational experiences [11, 12]. A study of more than 15,000 Chinese high school students showed that nearly 20% had suicidal thoughts, and more than two-thirds felt pressured by intense study [13]. Currently, most research on academic burnout in China focuses on nurses, high school and medical students, and college students in the liberal arts, physical education, teaching, engineering, and other specialties. Although relevant research has been gaining attention, it remains somewhat limited. In addition, survey research on academic burnout among college students in Heilongjiang Province is lacking. This study evaluated the level of academic burnout among college students and its association with influencing factors and proposes intervention strategies to promote the development of students’ physical and mental health. Furthermore, it provides a theoretical basis for the implementation of preventive interventions against academic burnout, which will be valuable to parents, college administrators, and educators.

## Methods

### Study design and participants

From September to December 2021, a cross-sectional study was conducted among college students in Heilongjiang Province using an online questionnaire survey with respondent-driven sampling. Respondent-driven sampling first selects a group of objects that meet the inclusion criteria as seeds. The seeds recommend qualified partners to enter the study, forming a first-class sampling population. When the sampling population reaches levels 5–6, the sample can better represent the population. First, we selected 13 universities in Heilongjiang Province using purposive sampling. Second, we selected 400 college students who met the inclusion criteria at each university. Finally, we selected 26,000 college students across the five levels for our investigation. We recovered 24,890 questionnaires. However, owing to the poor quality of some questionnaires, 22,983 valid questionnaires were included in the final analysis, with an effective recovery rate of 88.40%.

The inclusion criteria were as follows: (1) students currently studying at colleges and universities; (2) informed consent and willingness to participate voluntarily in this research; and (3) the ability to express their opinions clearly and with a good orientation to time, place, and people. Individuals who reported a previous mental illness or disturbance of consciousness were excluded from the study.

### Questionnaire

The questionnaire included a general situation questionnaire and an academic burnout survey for college students.

### General information questionnaire

The general situation questionnaire included the investigation of gender (male and female), place of origin (urban and non-urban), grade (freshman, sophomore, junior year, senior year, fifth year of college, master, doctor), whether an only child (only son, non-only child), residential experience in middle and high school, the highest level of education received by his or her parents (primary school and below, junior high school, high school or technical secondary school, junior college, undergraduate, master degree and above), monthly living expenses (< 1,000 yuan, 1,000–1,500 yuan, 1,500–2,000 yuan, 2,500–3,000 yuan, > 3,000 yuan), the pressure of study and life in the past two months (1-extremely little stressed, 2-very little stressed, 3-little stressed,4-much stressed, 5-very much stressed, 6-extremely much stressed), the degree of interest in professional knowledge (very interested, have interest, generally, less interest, no interest), weekly exercise time (1 h, 2 h, 3 h, 4 h), and smoking (smoking, quit smoking, never smoked), drinking (drinking, quit drinking, never drank), overall satisfaction with study and life (very satisfied, satisfied, generally, dissatisfied,very dissatisfied), and sleep quality in the past two months (very bad, pretty bad, generally, good, very good).

Investigation on academic burnout of college students.

The survey of college students’ academic burnout adopted the MBI-GS [5]. The scale was divided into three dimensions: emotional exhaustion (five items), cynicism (four items), and reduced personal accomplishment (six items). These items were rated on a seven-point Likert scale (0 represents ‘never’, 1 represents ‘rarely’, 2 represents ‘occasionally’, 3 represents ‘general’, 4 represents ‘often’, 5 represents ‘frequent’, and 6 represents ‘everyday’). The score was divided by 15 to obtain an average score, and the average score was multiplied by 20 (converted to 100 standard scores). A score below 50 indicates a good working condition, 50–75 indicates a certain degree of burnout, 75–100 indicates severe burnout and more than 100 indicates very serious burnout. The reliability of the study scale was confirmed using Cronbach’s alpha with 0.89 for emotional exhaustion, 0.87 for cynicism, and 0.79 for reduced personal accomplishment.

### Data analysis

SPSS Version 25.0 was used for the statistical analysis. General demographic data were summarised using descriptive statistics. Enumeration data were expressed as n (%), and measurement data were expressed as mean ± standard deviation (SD). A normality test was performed on each scale score. A independent sample t-test or one-way analysis of variance was used to compare the academic burnout scores of students with different demographic characteristics. Homogeneity of variances was also tested during one-way analysis of variance. LSD method was adopted to make multiple comparisons for meaningful indicators of one-way analysis of variance. The risk factors for burnout among students were analysed using multiple linear regression. The total academic burnout score was used as the dependent variable, and the sociodemographic characteristics of the respondents were used as independent variables. *P* < 0.05 indicated that differences were statistically significant.

## Results

### Sociodemographic characteristics of respondents

A total of 22,983 students participated in the survey, including 12,217 males (53.2%) and 10,766 females (46.8%). A total of 13,721 (59.7%) students were from urban areas, and 52.5% of the students were only children. More than half (*n* = 12,633, 55.0%) of the students were in their first year of university, and 60.6% of the students did not hold any position at school. Most parents’ highest education level was junior high school (*n* = 8,012, 34.9%). Among the participants, 93.3% had never smoked, 71.5% never drank alcohol, 55.3% exercised at least once a week, and 38.8% reported good sleep quality. Most students (*n* = 9,755, 42.4%) reported living on a budget of approximately 1,000–1,500 yuan per month.

Notably, only 9.5% of the respondents said that the impact of studying and life pressures in the past two months was minimal. Only 40.2% of the respondents expressed interest in the professional knowledge they were currently acquiring. Almost 40% of the respondents reported satisfaction with their studies and lives (*n* = 9,077, 39.5%). Table 1 shows the basic respondent information.

**Table 1:** Sociodemographic information of the participants

**Variable**		**Number**	**Percentage (%)**
Gender	Male	12,217	53.2
	Female	10,766	46.8
Place of origin	Urban	13,721	59.7
	Non-urban	9,262	40.3
Grade	Freshman	12,633	55.0
	Sophomore	5,789	25.2
	Junior year	2,651	11.5
	Senior year	794	3.5
	Fifth year of college	30	0.1
	Master	933	4.1
	Doctor	153	0.6
Whether an only child	Only son	12,077	52.5
	Non-only child	10,906	47.5
Whether you hold a position in the university	Student leaders (class/ student/society, etc.)	7,850	34.2
	No job	13,922	60.6
	Student leader in the past	1,211	5.2
Residential experience in middle and high school	Yes	13,469	58.6
	No	9,514	41.4
The highest level of education received by his or her parents	Primary school and below	1,434	6.2
	Junior high school	8,012	34.9
	High school or technical secondary school	7,048	30.7
	Junior college	2,526	11.0
	Undergraduate	3,476	15.1
	Master degree or above	487	2.1
Monthly living expenses(Yuan)	< 1,000	1,409	6.1
	1,000–1,500	9,755	42.4
	1,500–2,000	8,121	35.3
	2,500–3,000	2,655	11.6
	> 3,000	1,043	4.6
The pressure of study and life in the past two months	1-Extremely little stressed	2,177	9.5
	2-Very little stressed	4,217	18.3
	3-Little stressed	8,363	36.4
	4-Much stressed	6,122	26.6
	5-Very much stressed	1,497	6.5
	6-Extremely much stressed	607	2.7
The degree of interest in professional knowledge	Very interested	5,014	21.8
	Have interested	9,243	40.2
	Generally	5,216	22.7
	Less interest	3,019	13.1
	No interest	491	2.2
Weekly exercise time (hours)	1	12,709	55.3
	2	6,430	28.0
	3	2,152	9.4
	4	1,682	7.3
Smoking	Smoking	979	4.3
	Quit smoking	557	2.4
	Never smoked	21,447	93.3
Drinking	Drinking	5,569	24.2
	Quit drinking	986	4.3
	Never drank	16,428	71.5
Overall satisfaction with study	Very satisfied	3,569	15.5
	Satisfied	9,077	39.5
	Generally	8,823	38.4
	Dissatisfied	1,196	5.2
	Very dissatisfied	318	1.4
Sleep quality in the past two months	Very bad	431	1.9
	Pretty bad	1,696	7.4
	Generally	7,728	33.6
	Good	8,924	38.8
	Very good	4,204	18.3

### Academic burnout

The total score of the students’ academic burnout was 40.73 (± 10.12) points. The scores for the reduced personal accomplishment, emotional exhaustion, and cynicism were 23.63 (± 6.55), 11.20 (± 6.05), and 5.91 (± 5.31), respectively. Students with academic burnout accounted for 20.5% (4,731/22,983).

Table 2 analyzes the degree of burnout of all students. The study found that 40.01% of students had a good learning condition, 55.16% had a certain degree of academic burnout, 3.55% of students had a relatively severe degree of academic burnout, and 1.28% had a very severe degree of academic burnout.

**Table 2:** Analysis of academic burnout degree

Score	Burnout level	**N**	**Percentage (%)**
0–50	Good learning condition	9173	40.01
50–75	There is a certain degree of academic burnout	12,646	55.16
75–100	Academic burnout is relatively serious	813	3.55
More than 100 points	Academic burnout is very serious	294	1.28

### Comparison of reduced personal accomplishment scores with different demographic characteristics

Table 3 shows score comparisons for students’ reduced personal accomplishment organized by demographic characteristics. Students who drank and then chose to abstain had lower reduced personal accomplishments than those who never drank. Higher parental education levels were associated with longer weekly exercise times, better quality sleep over the past two months, more substantial overall satisfaction with learning and life in general, and higher reduced personal accomplishment scores. Students who were very interested in their current majors reported higher reduced personal accomplishment scores than other groups. The result of pairwise comparisons among different demographic variables in reduced personal accomplishment dimension (seen in Additional file 1).

**Table 3:** Comparison of reduced personal accomplishment scores with different demographic characteristics

Variable		n	Reduced personal accomplishment scores		t/F	*P*	Effect size
			Mean	SD			
Gender	Male	12,217	24.07	6.96	10.994	< 0.001	0.005
	Female	10,766	23.13	6.02			
Place of origin	Urban	13,721	23.97	6.71	9.808	0.101	0.004
	Non-urban	9,262	23.12	6.28			
Grade	Freshman	12,633	24.16	6.48	42.768	< 0.001	0.011
	Sophomore	5,789	22.62	6.38			
	Junior year	2,651	23.38	7.15			
	Senior year	794	22.67	6.44			
	Fifth year of college	30	20.87	7.88			
	Master	933	24.14	6.02			
	Doctor	153	24.42	6.23			
Whether an only child	Only son	12,077	23.97	6.80	8.362	< 0.001	0.003
	Non-only child	10,906	23.25	6.23			
Whether you hold a position in the university	Student leaders (class/ student/society, etc.)	7,850	24.49	6.55	10.974	< 0.001	0.009
	No job	13,922	23.15	6.50			
	Student leader in the past	1,211	23.64	6.60			
Residential experience in middle and high school	Yes	13,469	23.38	6.41	-6.756	< 0.001	0.002
	No	9,514	23.98	6.73			
The highest level of education received by his or her parents	Primary school and below	1,434	22.10	6.25	35.991	< 0.001	0.008
	Junior high school	8,012	23.28	6.29			
	High school or technical secondary school	7,048	23.75	6.53			
	Junior college	2,526	23.91	6.66			
	Undergraduate	3,476	24.45	6.90			
	Master degree and above	487	24.78	7.44			
Monthly living expenses(Yuan)	< 1,000	1,409	23.66	6.97	8.058	< 0.001	0.001
	1,000–1,500	9,755	23.40	6.42			
	1,500–2,000	8,121	23.67	6.47			
	2,500–3,000	2,655	24.17	6.56			
	> 3,000	1,043	23.98	7.61			
The pressure of study and life in the past two months	1-Extremely little stressed	2,177	23.66	6.97	8.058	< 0.001	0.066
	2-Very little stressed	4,217	23.41	6.42			
	3-Little stressed	8,363	23.67	6.47			
	4-Much stressed	6,122	24.17	6.56			
	5-Very much stressed	1,497	23.98	7.61			
	6-Extremely much stressed	607	23.66	6.97			
The degree of interest in professional knowledge	Very interested	5,014	28.04	6.70	82.919	< 0.001	0.171
	Have interested	9,243	23.80	5.68			
	Generally	5,216	20.82	5.57			
	Less interest	3,019	21.57	5.71			
	No interest	491	17.86	8.43			
Weekly exercise time (hours)	1	12,709	22.49	6.26	39.629	< 0.001	0.049
	2	6,430	24.28	6.20			
	3	2,152	25.71	6.64			
	4	1,692	27.03	7.63			
Smoking	Smoking	979	23.41	7.37	0.0685	0.504	0.001
	Quit smoking	557	23.50	6.94			
	Never smoked	21,447	23.64	6.50			
Drinking	Drinking	5,569	22.74	6.50	72.647	< 0.001	0.006
	Quit drinking	986	23.27	6.93			
	Never drank	16,428	23.95	6.52			
Overall satisfaction with study	Very satisfied	3,569	29.62	6.63	82.594	< 0.001	0.0241
	Satisfied	9,077	24.65	5.55			
	Generally	8,823	21.06	5.22			
	Dissatisfied	1,196	18.83	5.82			
	Very dissatisfied	318	16.76	9.83			
Sleep quality in the past two months	Very bad	431	20.08	8.51	50.018	< 0.001	0.080
	Pretty bad	1,696	21.06	6.48			
	Generally	7,728	22.13	5.99			
	Good	8,924	24.08	5.95			
	Very good	4,204	26.83	7.11			

### Comparison of emotional exhaustion scores with different demographic characteristics

Table 4 lists the results of a comparison of emotional exhaustion scores with different demographic characteristics. Female students who served as student leaders, were in the first year, had siblings, and were not living on campus for one month in middle and high school, had lower emotional exhaustion scores than other groups. Students whose parents had a master’s degree or above reported a monthly living cost of over 3,000 yuan, smoked and drank alcohol, and had higher emotional exhaustion scores. The result of pairwise comparisons among different demographic variables in emotional exhaustion dimension (seen in Additional file 2).

**Table 4:** Comparison of emotional exhaustion scores with different demographic characteristics

Variable		n	Emotional exhaustion scores		t/F	*P*	Effect size
			Mean	SD			
Gender	Male	12,217	11.28	6.39	2.367	0.018	0.001
	Female	10,766	11.09	5.65			
Place of origin	Urban	13,721	11.18	6.23	-0.321	0.748	0.001
	Non-urban	9,262	11.21	5.77			
Grade	Freshman	12,633	10.70	6.03	31.975	< 0.001	0.008
	Sophomore	5,789	11.83	5.95			
	Junior year	2,651	11.79	6.37			
	Senior year	794	11.92	5.82			
	Fifth year of college	30	13.50	6.00			
	Master	933	11.40	5.66			
	Doctor	153	11.54	6.23			
Whether an only child	Only son	12,077	11.11	6.34	-2.132	0.033	0.001
	Non-only child	10,906	11.28	5.72			
Whether you hold a position in the university	Student leaders (class/ student/society, etc.)	7,850	10.95	6.21	13.888	< 0.001	0.001
	No job	13,922	11.27	5.94			
	Student leader in the past	1,211	11.81	6.18			
Residential experience in middle and high school	Yes	13,469	11.27	5.85	2.310	0.021	0.001
	No	9,514	11.08	6.33			
The highest level of education received by his or her parents	Primary school and below	1,434	11.29	5.64	1.328	< 0.001	0.001
	Junior high school	8,012	11.17	5.73			
	High school or technical secondary school	7,048	11.11	6.06			
	Junior college	2,526	11.13	6.18			
	Undergraduate	3,476	11.34	6.62			
	Master degree and above	487	11.62	7.12			
Monthly living expenses(Yuan)	< 1,000	1,409	11.59	6.31	3.828	0.004	0.001
	1,000–1,500	9,755	11.11	5.84			
	1,500–2,000	8,121	11.16	6.02			
	2,500–3,000	2,655	11.19	6.35			
	> 3,000	1,043	11.69	6.99			
The pressure of study and life in the past two months	1-Extremely little stressed	2,177	7.59	7.30	9.084	< 0.001	0.114
	2-Very little stressed	4,217	9.21	5.27			
	3-Little stressed	8,363	11.07	5.23			
	4-Much stressed	6,122	12.59	5.48			
	5-Very much stressed	1,497	14.56	6.07			
	6-Extremely much stressed	607	17.19	8.57			
The degree of interest in professional knowledge	Very interested	5,014	8.76	6.87	77.225	< 0.001	0.077
	Have interested	9,243	10.82	5.40			
	Generally	5,216	12.91	5.29			
	Less interest	3,019	12.56	5.63			
	No interest	491	16.27	7.69			
Weekly exercise time (hours)	1	12,709	11.82	5.76	15.841	< 0.001	0.015
	2	6,430	10.69	5.92			
	3	2,152	10.09	6.19			
	4	1,692	9.79	7.68			
Smoking	Smoking	979	12.36	6.76	28.297	< 0.001	0.002
	Quit smoking	557	12.22	6.68			
	Never smoked	21,447	11.11	5.99			
Drinking	Drinking	5,569	12.11	5.98	10.568	< 0.001	0.001
	Quit drinking	986	11.98	6.46			
	Never drank	16,428	10.83	6.01			
Overall satisfaction with study	Very satisfied	3,569	8.06	7.42	73.342	< 0.001	0.113
	Satisfied	9,077	10.17	5.31			
	Generally	8,823	12.83	5.12			
	Dissatisfied	1,196	14.52	5.90			
	Very dissatisfied	318	11.75	8.24			
Sleep quality in the past two months	Very bad	431	16.22	7.27	48.760	< 0.001	0.078
	Pretty bad	1,696	13.99	5.69			
	Generally	7,728	12.47	5.44			
	Good	8,924	10.42	5.53			
	Very good	4,204	8.83	6.85			

### Comparison of cynicism scores with different demographic characteristics

Students whose parents’ highest level of education was primary school and lower, whose monthly living expenses were less than 1,000 yuan, who exercised less than one hour per week, and who had quit smoking or drinking scored higher on cynicism. See Table 5 for the other detailed results. The result of pairwise comparisons among different demographic variables in cynicism dimension (seen in Additional file 3).

**Table 5:** Comparison of cynicism scores with different demographic characteristics

Variable		n	Cynicism scores		t/F	*P*	Effect size
			Mean	SD			
Gender	Male	12,217	5.97	5.58	1.994	0.046	0.075
	Female	10,766	5.75	4.99			
Place of origin	Urban	13,721	5.75	5.37	-5.652	0.068	0.001
	Non-urban	9,262	6.15	5.21			
Grade	Freshman	12,633	5.29	5.12	69.612	< 0.001	0.018
	Sophomore	5,789	6.70	5.37			
	Junior year	2,651	6.88	5.74			
	Senior year	794	6.54	5.19			
	Fifth year of college	30	8.23	5.26			
	Master	933	6.09	5.16			
	Doctor	153	5.56	5.26			
Whether an only child	Only son	12,077	5.76	5.43	-4.454	< 0.001	0.001
	Non-only child	10,906	6.07	5.16			
Whether you hold a position in the university	Student leaders (class/ student/society, etc.)	7,850	5.47	5.22	44.807	< 0.001	0.004
	No job	13,922	6.09	5.32			
	Student leader in the past	1,211	6.58	5.58			
Residential experience in middle and high school	Yes	13,469	6.08	5.27	5.896	0.115	0.002
	No	9,514	5.66	5.36			
The highest level of education received by his or her parents	Primary school and below	1,434	6.65	5.22	7.853	< 0.001	0.002
	Junior high school	8,012	5.97	5.12			
	High school or technical secondary school	7,048	5.84	5.32			
	Junior college	2,526	5.61	5.23			
	Undergraduate	3,476	5.84	5.70			
	Master degree and above	487	5.70	5.92			
Monthly living expenses(Yuan)	< 1,000	1,409	6.42	5.62	6.775	< 0.001	0.001
	1,000–1,500	9,755	5.94	5.25			
	1,500–2,000	8,121	5.73	5.17			
	2,500–3,000	2,655	5.94	5.44			
	> 3,000	1,043	6.25	6.06			
The pressure of study and life in the past two months	1-Extremely little stressed	2,177	3.61	5.73	8.301	< 0.001	0.077
	2-Very little stressed	4,217	4.32	4.49			
	3-Little stressed	8,363	5.89	4.86			
	4-Much stressed	6,122	6.79	5.12			
	5-Very much stressed	1,497	8.19	5.68			
	6-Extremely much stressed	607	10.90	7.64			
The degree of interest in professional knowledge	Very interested	5,014	3.58	5.29	69.443	< 0.001	0.108
	Have interested	9,243	5.35	4.75			
	Generally	5,216	7.98	4.97			
	Less interest	3,019	7.03	5.06			
	No interest	491	11.16	6.86			
Weekly exercise time (hours)	1	12,709	6.46	5.22	10.670	< 0.001	0.014
	2	6,430	5.34	5.06			
	3	2,152	4.89	5.20			
	4	1,692	5.19	6.40			
Smoking	Smoking	979	7.19	6.16	53.852	< 0.001	0.005
	Quit smoking	557	7.38	6.13			
	Never smoked	21,447	5.81	55.23			
Drinking	Drinking	5,569	6.77	5.47	12.020	< 0.001	0.010
	Quit drinking	986	6.72	5.59			
	Never drank	16,428	5.57	5.20			
Overall satisfaction with study	Very satisfied	3,569	3.47	5.76	37.909	< 0.001	0.127
	Satisfied	9,077	4.68	4.54			
	Generally	8,823	7.49	4.89			
	Dissatisfied	1,196	9.25	5.03			
	Very dissatisfied	318	12.08	7.78			
Sleep quality in the past two months	Very bad	431	10.44	6.86	48.478	< 0.001	0.075
	Pretty bad	1,696	8.11	5.29			
	Generally	7,728	7.11	5.18			
	Good	8,924	5.12	4.73			
	Very good	4,204	4.01	5.44			

### Results of linear regression analysis of academic burnout among students

Table 6 displays that gender, grade, parents’ education, monthly living expenses, the degree of study and life pressure experienced over the past two months, interest in the current professional knowledge, sports, overall satisfaction with one’s studies and life influence student academic burnout. The adjusted r-square value is 0.108.

**Table 6:** Multiple linear regression analysis of risk factors for student academic burnout

Variable	Dummy variable	Nonstandardized coefficient	Standard error	Standardization coefficient	Effect size	t/F	*P*	Nonstandardized Coefficient 95%CI	
								Lower limit	Upper limit
Constant		44.75	0.93			47.99	< 0.001	42.93	46.58
Gender		-1.21	0.19	-0.05	0.01	-6.50	< 0.001	-1.58	-0.85
Place of origin		-0.26	0.20	-0.01	0.01	-1.33	0.19	-0.65	0.13
Grade	Sophomore	1.14	0.21	0.04	0.01	5.48	< 0.001	0.74	1.55
	Junior year	1.72	0.28	0.041	0.01	6.11	< 0.001	1.17	2.28
	Senior year	0.46	0.48	0.01	0.01	0.95	0.34	-0.48	1.39
	Fifth year of college	-0.36	2.34	-0.00	0.01	-0.15	0.88	-4.94	4.22
	Master	1.56	0.45	0.02	0.01	3.46	< 0.001	0.68	2.45
	Doctor	0.38	1.05	0.00	0.01	0.36	0.72	-1.67	2.43
Whether an only child		0.20	0.19	0.01	0.01	1.04	0.30	-0.18	0.57
Whether you hold a position in the university	Student leaders (class/ student/society, etc.)	0.29	0.19	0.01	0.01	1.58	0.12	-0.07	0.66
	Student leader in the past	0.59	0.41	0.01	0.01	1.45	0.15	-0.21	1.39
Residential experience in middle and high school		0.09	0.18	0.00	0.01	0.48	0.63	-0.27	0.45
The highest level of education received by his or her parents		0.43	0.08	0.04	0.01	5.40	< 0.001	0.27	0.58
Monthly living expenses(Yuan)		0.20	0.10	0.01	0.002	1.96	0.05	0	0.39
The pressure of study and life in the past two months		1.19	0.09	0.10	0.05	13.76	< 0.001	1.02	1.36
The degree of interest in professional knowledge		0.26	0.09	0.02	0.01	2.81	0.01	0.08	0.44
Weekly exercise time (hours)		0.51	0.10	0.04	0.01	5.18	0.11	0.32	0.74
Smoking	Smoking	1.08	0.44	0.02	0.01	2.44	0.02	0.21	1.94
	Quit smoking	1.78	0.57	0.02	0.01	3.11	< 0.001	0.66	2.91
Drinking	Drinking	-0.30	0.44	-0.00	0.01	-0.67	0.50	-1.16	0.57
	Quit drinking	4.13	0.11	0.28	0.01	37.69	0.00	3.92	4.35
Overall satisfaction with study		-1.17	0.12	-0.07	0.01	-9.58	< 0.001	-1.41	-0.93
Sleep quality in the past two months		-0.01	0.11	-0.00	0.02	-0.08	0.93	-0.22	0.20

## Discussion

This cross-sectional study explored academic burnout levels and the factors influencing them among university students. Previous studies have shown that academic burnout presents a dilemma between not wanting to learn and not wanting to give up completely. Unlike learning boredom, academic burnout is reversible [14–16]. If detected and addressed promptly, burnout can be relieved. Burnout reduction interventions can help students embark on a path of enjoying learning from a declining state of weariness. Therefore, research on academic burnout is critical.

### Analysis of academic burnout levels

We found that more than half of the college students who participated in the survey had academic burnout: 55.16% had mild burnout, 3.55% had serious burnout, and 1.28% had very serious burnout. Previous studies have shown that students with academic burnout accounted for 27% ~ 75% of all students [17, 18].

Factors affecting academic burnout include gender, grade, parents’ education level, monthly living expenses, the pressure of study and life in the past two months,the degree of interest in professional knowledge, smoking.

Studies on the relationship between gender and academic burnout presented varied results [19–22]. Men had higher burnout scores across all dimensions than women. This finding was inconsistent with the finding that women had significantly higher burnout scores [23]. The results of our study may reflect the differences in gender roles and achievement motivation between men and women. In college, men frequently exhibit stronger achievement expectations than women, a more robust sense of competition, and more intense achievement self-evaluation. Thus, when men’s achievement expectations are not met, they exhibit more negative emotions, avoidant behaviours, and a lower sense of achievement.

In terms of grades, doctoral, master’s, and first-year university students had higher levels of reduced personal accomplishment and lower emotional exhaustion and cynicism scores. This result suggests that they have more mental energy for learning. Freshmen who have just entered university have not fully adapted to life and studies at the university, so they maintain the same enthusiasm for learning that they had in high school. At the same time, they are full of expectations and have a longing for university life. In a new learning environment, classmates and teachers may inspire students to express themselves more effectively. From the perspective of the established curriculum, many courses are available to freshmen. They are required to take basic public and professional courses. Therefore, they were not relaxed about their studies. Sophomore, junior, and senior students gradually become familiar with the school environment after more than one year of study, but they also found that the university is not as pleasant as they had imagined. Therefore, disappointment may be expressed emotionally. Some students were disappointed with their school, while others were dissatisfied with their studies and gradually shifted their interests to other areas. Some individuals exhibit destructive behaviours, skipping or not attending classes and not handing in assignments. The results of this study are consistent with those of previous ones [24–26].

This study found that students who never smoke experienced a greater reduction in personal accomplishment. Students who never smoke had significantly lower emotional exhaustion and cynicism scores. College students may choose to smoke when they encounter problems and difficulties in their studies and daily lives. However, prolonged smoke consumption causes psychological problems and creates a vicious circle. Other studies have also confirmed this phenomenon [27–29].

This study found that students with monthly living expenses of 2500–3000 yuan had the highest levels of reduced personal accomplishment. Excessively high or low monthly living expenses can lead to emotional exhaustion and high cynicism scores. Students experiencing poor economic conditions tend to have inferiority complexes. They may have more trouble meeting academic expenses, leading to burnout. In contrast, students with monthly living expenses of more than 3000 yuan respond to the rapid changes in contemporary society’s consumption structure with the development of the social economy. The consumption of college students has subtly changed. To meet their growing material needs or for reasons of vanity and social comparisons, they form unhealthy consumption habits. Some college students exhibit serious excessive and advanced consumption which may lead to psychological problems [30, 31].

The work demand resource theory first pointed out that work resources can buffer the negative impact of work on stress [32]. Social support, autonomy, and performance feedback can help employees cope with high work requirements and prevent burnout and other health problems. This suggests that we should constantly improve the social support system of college students. Specifically, college students’ family members should try to maintain the stability of the family structure and good economic status. College students’ friends, counsellors, teachers, and others should pay more attention to college students, form interactive relationships, and enable them to obtain spiritual and material support. Second, work-demand-resource theory shows that employees who actively participate in work are motivated to maintain participation. In the context of college students when learning requirements become too high or they lack work resources, they should learn to use personal initiatives to change their environments. For example, they can actively ask for feedback on their performance, voluntarily participate in new projects, or strengthen their cooperation with others in a learning environment. By carefully designing their learning conditions and improving their work needs and resources, they can create a better match between the people and their environments. Third, the work demand-resource-theory indicates that employees with high work requirements may be exhausted and enter a loss cycle. This indicates that we should focus more on the senior undergraduate group. They often have higher self-requirements and face heavier employment pressures, making them more likely to accumulate fatigue and lead to self-destructive behaviours such as making mistakes, avoiding, and having conflicts with classmates. Society, schools, and parents should give them more attention, resources, and support, which can help them reduce academic burnout and pressure and avoid self-destructive behaviours caused by high academic burnout.

### Limitations

Our study has some limitations. The cross-sectional design is a limitation of this study. However, we could not establish a causal relationship among the identified associations. Therefore, longitudinal studies are necessary to establish variables that show true causal relationships with burnout syndrome in this population. In addition, we used an Internet survey owing to the COVID-19 pandemic. The reliability and validity of the survey results may have been affected by participants’ responses. Our study included college students from Heilongjiang Province, and the overall sample size was large and representative. Therefore, a nationwide investigation should be conducted in the future.

## Conclusions

More than half the students that were part of the survey had academic burnout. Several factors affect academic burnout. Implementing an effective wellness program may be sufficient to reduce student burnout. We recommend that all students undergo an annual long-term burnout assessment to determine the effectiveness of the wellness program and modify it accordingly.

## Supplementary Information

**Additional file 1.** The result of pairwise comparisons among different demographic variables in emotional exhaustion dimension.

**Additional file 2.** The result of pairwise comparisons among different demographic variables in cynicism dimension.

**Additional file 3.** The result of pairwise comparisons among different demographic variables in reduced personal accomplishment dimension.

## Data Availability

The data set used and/or analyzed in the current study may be obtained from the corresponding author upon reasonable request.

## Abbreviation

MBI-GS: The Maslach Burnout Inventory General Survey
