# Supplementary material for: Factors associated with academic burnout and its prevalence among university students: a cross-sectional study
Source: BMC Med Educ. 2023 May 6;23:317. doi: 10.1186/s12909-023-04316-y (PMC10163855; doi:10.1186/s12909-023-04316-y)
Supplement: Supplementary file 1 — Additional file 1. The result of pairwise comparisons among different demographic variables in emotional exhaustion dimension. [file 12909_2023_4316_MOESM1_ESM.docx]

**Additional file 1:The result of pairwise comparisons among different demographic variables in emotional exhaustion dimension**

| **Variable** |  | **Mean rank diff** | **Significant** | **Summary** | **Adjusted P Value** |
| --- | --- | --- | --- | --- | --- |
| Grade | Freshman VS. sophomore | -1.12883* | Yes | **** | P<0.0001 |
|  | Freshman VS. junior | -1.08320* | Yes | **** | P<0.0001 |
|  | Freshman VS. senior | -1.22025* | Yes | **** | P<0.0001 |
|  | Freshman VS. senior | -2.79708* | Yes | * | 0.011 |
|  | Freshman VS. Master | -.69365* | Yes | *** | 0.001 |
|  | Freshman VS Doctor | -0.83303 | No | ns | 0.089 |
|  | Sophomore VS. junior | 0.04563 | No | ns | 0.747 |
|  | Sophomore VS. senior | -0.09142 | No | ns | 0.689 |
|  | Sophomore VS. senior | -1.66825 | No | ns | 0.130 |
|  | Sophomore VS Master | .43518* | Yes | * | 0.041 |
|  | Sophomore VS Doctor | 0.2958 | No | ns | 0.549 |
|  | Junior VS. senior | -0.13706 | No | ns | 0.574 |
|  | Junior VS. Senior | -1.71388 | No | ns | 0.121 |
|  | Junior VS Master | 0.38955 | No | ns | 0.090 |
|  | Junior VS Doctor | 0.25017 | No | ns | 0.618 |
|  | Senior VS. Senior | -1.57683 | No | ns | 0.160 |
|  | Senior VS Master | 0.5266 | No | ns | 0.070 |
|  | Senior VS Doctor | 0.38723 | No | ns | 0.467 |
|  | Fifth year of college VS. Master's | 2.10343 | No | ns | 0.060 |
|  | Fifth year of college VS Doctor | 1.96405 | No | ns | 0.103 |
|  | Master VS Doctor | -0.13938 | No | ns | 0.791 |
| Whether you hold a position in the university | Student leaders (class/ student/society, etc.) VS No job | -.32364* | Yes | **** | P<0.0001 |
|  | Student leader (class/Student union/club, etc.) VS Student leader in the past | -.86058* | Yes | **** | P<0.0001 |
|  | No job VS. Student leader in the past | .32364* | Yes | **** | P<0.0001 |
| The highest level of education received by his or her parents | Junior high school and below VS junior high school | 0.11711 | No | ns | 0.500 |
|  | Junior high school and below VS High school or technical secondary school school | 0.18056 | No | ns | 0.303 |
|  | Junior high VS junior high | 0.15669 | No | ns | 0.434 |
|  | Junior high VS Undergraduate | -0.05254 | No | ns | 0.782 |
|  | Junior high school VS Master's and above | -0.33413 | No | ns | 0.292 |
|  | Junior high school VS. high school or technical school | 0.06346 | No | ns | 0.521 |
|  | Junior high school VS junior college | 0.03958 | No | ns | 0.774 |
|  | Junior high school VS Undergraduate | -0.16964 | No | ns | 0.167 |
|  | Junior high school VS Master's degree or above | -0.45124 | No | ns | 0.110 |
|  | High school or technical secondary school VS. junior college | -0.02388 | No | ns | 0.865 |
|  | High school or technical secondary school school VS Undergraduate | -0.2331 | No | ns | 0.063 |
|  | High school or technical secondary school school VS Master's degree or above | -0.5147 | No | ns | 0.069 |
|  | Junior college VS Undergraduate | -0.20922 | No | ns | 0.186 |
|  | Junior college VS Master degree or above | -0.49082 | No | ns | 0.101 |
|  | Undergraduate VS Master degree or above | -0.28159 | No | ns | 0.336 |
| Monthly living expenses(Yuan) | <1,000 VS1,000-1,500 | .48093* | Yes | ** | 0.005 |
|  | <1,000 VS1,500-2,000 | .42828* | Yes | * | 0.014 |
|  | <1,000 VS2,500-3,000 | .39740* | Yes | * | 0.046 |
|  | <1,000 VS>3,000 | -0.10387 | No | ns | 0.674 |
|  | 1,000-1,500 VS 1,500-2,000 | -0.05265 | No | ns | 0.562 |
|  | 1,000-1,500 VS 2,500-3,000 | -0.08353 | No | ns | 0.528 |
|  | 1,000-1,500 VS>3,000 | -.58480* | Yes | ** | 0.003 |
|  | 1,500-2,000 VS 2,500-3,000 | -0.03088 | No | ns | 0.819 |
|  | 1,500-2,000 VS>3,000 | -.53216* | Yes | ** | 0.007 |
|  | 2,500-3,000 VS>3,000 | -.50127* | Yes | * | 0.023 |
| The pressure of study and life in the past two months | 1-Extremely little stressed VS2-Very little stressed | -1.62114* | Yes | **** | P<0.0001 |
|  | 1-Extremely little stressed VS3-Little stressed | -3.47990* | Yes | **** | P<0.0001 |
|  | 1-Extremely little stressed VS4-Much stressed | -5.00570* | Yes | **** | P<0.0001 |
|  | The pressure is minimal VS5-Very much stressed | -6.97454* | Yes | **** | P<0.0001 |
|  | 1-Extremely little stressed VS 6-Extremely much stressed | -9.60781* | Yes | **** | P<0.0001 |
|  | 2-Very little stressedVS3-Little stressed | -1.85875* | Yes | **** | P<0.0001 |
|  | 2-Very little stressedVS4-Much stressed | -3.38456* | Yes | **** | P<0.0001 |
|  | 2-Very little stressedVS5-Very much stressed | -5.35339* | Yes | **** | P<0.0001 |
|  | 2-Very little stressed VS 6-Extremely much stressed | -7.98667* | Yes | **** | P<0.0001 |
|  | 3-Little stressedVS4-Much stressed | -1.52581* | Yes | **** | P<0.0001 |
|  | 3-Little stressedVS5-Very much stressed | -3.49464* | Yes | **** | P<0.0001 |
|  | 3-Little stressed VS 6-Extremely much stressed | -6.12792* | Yes | **** | P<0.0001 |
|  | 4-Much stressedVS5-Very much stressed | -1.96883* | Yes | **** | P<0.0001 |
|  | 4-Much stressed VS6-Extremely much stressed | -4.60211* | Yes | **** | P<0.0001 |
|  | 5-Very much stressed VS 6-Extremely much stressed | -2.63328* | Yes | **** | P<0.0001 |
| The degree of interest in professional knowledge | Very interested VS Have interested | -2.05359* | Yes | **** | P<0.0001 |
|  | Very interested VS. Generally | -4.14556* | Yes | **** | P<0.0001 |
|  | Very interested VS Less interest | -3.79572* | Yes | **** | P<0.0001 |
|  | Very interested VS No interest | -7.50482* | Yes | **** | P<0.0001 |
|  | Have interested VS. Generally | -2.09197* | Yes | **** | P<0.0001 |
|  | Have interested VS are Less interest | -1.74213* | Yes | **** | P<0.0001 |
|  | Have interested VS. No interest | -5.45123* | Yes | **** | P<0.0001 |
|  | General VS is Less interest | .34984* | Yes | ** | 0.009 |
|  | Generally VS No interest | -3.35926* | Yes | **** | P<0.0001 |
|  | Less interest VS No interest | -3.70910* | Yes | **** | P<0.0001 |
| Weekly exercise time (hours) | 1 hour VS2 hours | 1.12461* | Yes | **** | P<0.0001 |
|  | 1 hour VS3 hours | 1.72994* | Yes | **** | P<0.0001 |
|  | 1 hour VS4 hours | 2.02994* | Yes | **** | P<0.0001 |
|  | 2 hours VS3 hours | .60533* | Yes | **** | P<0.0001 |
|  | 2 hours VS4 hours | .90533* | Yes | **** | P<0.0001 |
|  | 3 hours VS4 hours | 0.3 | No | ns | 0.124 |
| Smoking | Smoking VS Quit smoking | 0.13795 | No | ns | 0.667 |
|  | Smoking VS Never smoked | 1.24899* | Yes | **** | P<0.0001 |
|  | Quit smoking VS Never smoked | 1.11104* | Yes | **** | P<0.0001 |
| Drinking | Drinking VS Quit Drinking | 0.13317 | No | ns | 0.522 |
|  | Drinking VS Never drank | 1.27933* | Yes | **** | P<0.0001 |
|  | Quit drinking VS Never drank | 1.14616* | Yes | **** | P<0.0001 |
| Overall satisfaction with study | Very satisfied VS Satisfied | -2.10064* | Yes | **** | P<0.0001 |
|  | Very satisfied VS Generally | -4.76028* | Yes | **** | P<0.0001 |
|  | Very satisfied VS Dissatisfied | -6.45534* | Yes | **** | P<0.0001 |
|  | Very satisfied VS Very dissatisfied | -9.68370* | Yes | **** | P<0.0001 |
|  | Satisfied VS Generally | -2.65964* | Yes | **** | P<0.0001 |
|  | Satisfied VS Dissatisfied | -4.35470* | Yes | **** | P<0.0001 |
|  | Satisfied VS very Very dissatisfied | -7.58306* | Yes | **** | P<0.0001 |
|  | Generally VS Dissatisfied | -1.69506* | Yes | **** | P<0.0001 |
|  | Generally VS Very dissatisfied | -4.92342* | Yes | **** | P<0.0001 |
|  | Dissatisfied VS Very dissatisfied | -3.22836* | Yes | **** | P<0.0001 |
| Sleep quality in the past two months | Very bad VS pretty bad | 2.22993* | Yes | **** | P<0.0001 |
|  | Very poor VS average | 3.74256* | Yes | **** | P<0.0001 |
|  | Very bad VS good | 5.79332* | Yes | **** | P<0.0001 |
|  | Very bad VS very good | 7.38918* | Yes | **** | P<0.0001 |
|  | Pretty bad VS so-so | 1.51263* | Yes | **** | P<0.0001 |
|  | Pretty bad VS pretty good | 3.56339* | Yes | **** | P<0.0001 |
|  | Pretty bad VS very good | 5.15926* | Yes | **** | P<0.0001 |
|  | VS in general is fine | 2.05076* | Yes | **** | P<0.0001 |
|  | VS in general is very good | 3.64662* | Yes | **** | P<0.0001 |
|  | Good VS very good | 1.59586* | Yes | **** | P<0.0001 |

****：P<0.001,***:P<0.001,**:P<0.01;*:P<0.05

ns: no significant
