# Supplementary material for: Factors associated with academic burnout and its prevalence among university students: a cross-sectional study
Source: BMC Med Educ. 2023 May 6;23:317. doi: 10.1186/s12909-023-04316-y (PMC10163855; doi:10.1186/s12909-023-04316-y)
Supplement: Supplementary file 2 — Additional file 2. The result of pairwise comparisons among different demographic variables in cynicism dimension. [file 12909_2023_4316_MOESM2_ESM.docx]

**Additional file 2:The result of pairwise comparisons among different demographic variables in cynicism dimension**

| **Variable** |  | **Mean rank diff** | **Significant** | **Summary** | **Adjusted P Value** |
| --- | --- | --- | --- | --- | --- |
| Grade | Freshman VS. sophomore | -1.41648* | Yes | **** | P<0.0001 |
|  | Freshman VS. junior | -1.59242* | Yes | **** | P<0.0001 |
|  | Freshman VS. senior | -1.25368* | Yes | **** | P<0.0001 |
|  | Freshman VS. senior | -2.94797* | Yes | ** | 0.002 |
|  | Freshman VS. Master | -.80681* | Yes | **** | P<0.0001 |
|  | Freshman VS Doctor | -0.27673 | No | ns | 0.518 |
|  | Sophomore VS. junior | -0.17593 | No | ns | 0.154 |
|  | Sophomore VS. senior | 0.16281 | No | ns | 0.414 |
|  | Sophomore VS. senior | -1.53149 | No | ns | 0.112 |
|  | Sophomore VS Master | .60967* | Yes | *** | 0.001 |
|  | Sophomore VS Doctor | 1.13976* | Yes | ** | 0.008 |
|  | Junior VS. senior | 0.33874 | No | ns | 0.112 |
|  | Junior VS. Senior | -1.35555 | No | ns | 0.161 |
|  | Junior VS Master | .78561* | Yes | **** | P<0.0001 |
|  | Junior VS Doctor | 1.31569* | Yes | ** | 0.003 |
|  | Senior VS. Senior | -1.69429 | No | ns | 0.083 |
|  | Senior VS Master | 0.44687 | No | ns | 0.079 |
|  | Senior VS Doctor | .97695* | Yes | * | 0.036 |
|  | Fifth year of college VS. Master's | 2.14116* | Yes | * | 0.028 |
|  | Fifth year of college VS Dr | 2.67124* | Yes | * | 0.011 |
|  | Master VS Doctor | 0.53008 | No | ns | 0.248 |
| Whether you hold a position in the university | Student leaders (class/ student/society, etc.) VS No job | -.62246* | Yes | **** | P<0.0001 |
|  | Student leader (class/Student union/club, etc.) VS Student leader in the past | -1.10403* | Yes | **** | P<0.0001 |
|  | No job VS. used to be a student leader in college | -.48157* | Yes | ** | 0.002 |
|  | Student leader (class/Student union/club, etc.) VS Student leader in the past | -1.10403* | Yes | **** | P<0.0001 |
| The highest level of education received by his or her parents | Junior high school and below VS junior high school | .68477* | Yes | **** | P<0.0001 |
|  | Junior high school and below VS High school or technical secondary school school | .80797* | Yes | **** | P<0.0001 |
|  | Junior high VS junior high | 1.03652* | Yes | **** | P<0.0001 |
|  | Junior high VS Undergraduate | .81416* | Yes | **** | P<0.0001 |
|  | Junior high school VS Master's and above | .95317* | Yes | *** | 0.001 |
|  | Junior high school VS. high school or technical school | 0.12319 | No | ns | 0.155 |
|  | Junior high school VS junior college | .35174* | Yes | ** | 0.004 |
|  | Junior high school VS Undergraduate | 0.12938 | No | ns | 0.230 |
|  | Junior high school VS Master's degree or above | 0.2684 | No | ns | 0.278 |
|  | High school or technical secondary school VS. junior college | 0.22855 | No | ns | 0.063 |
|  | High school or technical secondary school school VS Undergraduate | 0.00619 | No | ns | 0.955 |
|  | High school or technical secondary school school VS Master's degree or above | 0.14521 | No | ns | 0.559 |
|  | Junior college VS Undergraduate | -0.22236 | No | ns | 0.109 |
|  | Junior college VS Master degree or above | -0.08335 | No | ns | 0.751 |
|  | Undergraduate VS Master's or above | 0.13902 | No | ns | 0.588 |
| Monthly living expenses(Yuan) | <1,000 VS1,000-1,500 | .48859* | Yes | *** | 0.001 |
|  | <1,000 VS1,500-2,000 | .69264* | Yes | **** | P<0.0001 |
|  | <1,000 VS2,500-3,000 | .48736* | Yes | ** | 0.005 |
|  | <1,000 VS>3,000 | 0.16963 | No | ns | 0.434 |
|  | 1,000-1,500 VS 1,500-2,000 | .20404* | Yes | ** | 0.010 |
|  | 1,000-1,500 VS 2,500-3,000 | -0.00124 | No | ns | 0.992 |
|  | 1,000-1,500 VS>3,000 | -0.31896 | No | ns | 0.065 |
|  | 1,500-2,000 VS 2,500-3,000 | -0.20528 | No | ns | 0.084 |
|  | 1,500-2,000 VS>3,000 | -.52301* | Yes | ** | 0.003 |
|  | 2,500-3,000 VS>3,000 | -0.31773 | No | ns | 0.101 |
| The pressure of study and life in the past two months | 1-Extremely little stressed VS2-Very little stressed | -.70836* | Yes | **** | P<0.0001 |
|  | 1-Extremely little stressed VS3-Little stressed | -2.27106* | Yes | **** | P<0.0001 |
|  | 1-Extremely little stressed VS4-Much stressed | -3.17922* | Yes | **** | P<0.0001 |
|  | The pressure is minimal VS5-Very much stressed | -4.57156* | Yes | **** | P<0.0001 |
|  | 1-Extremely little stressed VS 6-Extremely much stressed | -7.28701* | Yes | **** | P<0.0001 |
|  | 2-Very little stressedVS3-Little stressed | -1.56270* | Yes | **** | P<0.0001 |
|  | 2-Very little stressedVS4-Much stressed | -2.47086* | Yes | **** | P<0.0001 |
|  | 2-Very little stressedVS5-Very much stressed | -3.86320* | Yes | **** | P<0.0001 |
|  | 2-Very little stressed VS 6-Extremely much stressed | -6.57865* | Yes | **** | P<0.0001 |
|  | 3-Little stressedVS4-Much stressed | -.90816* | Yes | **** | P<0.0001 |
|  | 3-Little stressedVS5-Very much stressed | -2.30050* | Yes | **** | P<0.0001 |
|  | 3-Little stressed VS 6-Extremely much stressed | -5.01594* | Yes | **** | P<0.0001 |
|  | 4-Much stressedVS5-Very much stressed | -1.39234* | Yes | **** | P<0.0001 |
|  | 4-Much stressed VS6-Extremely much stressed | -4.10779* | Yes | **** | P<0.0001 |
|  | 5-Very much stressed VS 6-Extremely much stressed | -2.71545* | Yes | **** | P<0.0001 |
| The degree of interest in professional knowledge | Very interested VS Have interested | -1.77532* | Yes | **** | P<0.0001 |
|  | Very interested VS. Generally | -4.40766* | Yes | **** | P<0.0001 |
|  | Very interested VS Less interest | -3.45508* | Yes | **** | P<0.0001 |
|  | Very interested VS No interest | -7.58555* | Yes | **** | P<0.0001 |
|  | Have interested VS. Generally | -2.63235* | Yes | **** | P<0.0001 |
|  | Have interested VS are Less interest | -1.67976* | Yes | **** | P<0.0001 |
|  | Have interested VS. No interest | -5.81023* | Yes | **** | P<0.0001 |
|  | General VS is Less interest | .95258* | Yes | **** | P<0.0001 |
|  | Generally VS No interest | -3.17789* | Yes | **** | P<0.0001 |
|  | Less interest VS No interest | -4.13047* | Yes | **** | P<0.0001 |
| Weekly exercise time (hours) | 1 hour VS2 hours | 1.12513* | Yes | **** | P<0.0001 |
|  | 1 hour VS3 hours | 1.57816* | Yes | **** | P<0.0001 |
|  | 1 hour VS4 hours | 1.27933* | Yes | **** | P<0.0001 |
|  | 2 hours VS3 hours | .45304* | Yes | *** | 0.001 |
|  | 2 hours VS4 hours | 0.1542 | No | ns | 0.284 |
|  | 3 hours VS4 hours | -0.29884 | No | ns | 0.081 |
| Smoking | Smoking VS Quit smoking | -0.1896 | No | ns | 0.500 |
|  | Smoking VS Never smoked | 1.38027* | Yes | **** | P<0.0001 |
|  | Quit smoking VS Never smoked | 1.56987* | Yes | **** | P<0.0001 |
| Drinking | Drinking VS Quit Drinking | 0.05533 | No | ns | 0.762 |
|  | Drinking VS Never drank | 1.20368* | Yes | **** | P<0.0001 |
|  | Quit drinking VS Never drank | 1.14836* | Yes | **** | P<0.0001 |
| Overall satisfaction with study | Very satisfied VS Satisfied | -1.20786* | Yes | **** | P<0.0001 |
|  | Very satisfied VS Generally | -4.01842* | Yes | **** | P<0.0001 |
|  | Very satisfied VS Dissatisfied | -5.78125* | Yes | **** | P<0.0001 |
|  | Very satisfied VS Very dissatisfied | -8.61070* | Yes | **** | P<0.0001 |
|  | Satisfied VS Generally | -2.81057* | Yes | **** | P<0.0001 |
|  | Satisfied VS Dissatisfied | -4.57339* | Yes | **** | P<0.0001 |
|  | Satisfied VS very Very dissatisfied | -7.40284* | Yes | **** | P<0.0001 |
|  | Generally VS Dissatisfied | -1.76282* | Yes | **** | P<0.0001 |
|  | Generally VS Very dissatisfied | -4.59227* | Yes | **** | P<0.0001 |
|  | Dissatisfied VS Very dissatisfied | -2.82945* | Yes | **** | P<0.0001 |
| Sleep quality in the past two months | Very bad VS pretty bad | 2.33297* | Yes | **** | P<0.0001 |
|  | Very poor VS average | 3.32801* | Yes | **** | P<0.0001 |
|  | Very bad VS good | 5.31525* | Yes | **** | P<0.0001 |
|  | Very bad VS very good | 6.42852* | Yes | **** | P<0.0001 |
|  | Pretty bad VS so-so | .99504* | Yes | **** | P<0.0001 |
|  | Pretty bad VS pretty good | 2.98228* | Yes | **** | P<0.0001 |
|  | Pretty bad VS very good | 4.09555* | Yes | **** | P<0.0001 |
|  | VS in general is fine | 1.98724* | Yes | **** | P<0.0001 |
|  | VS in general is very good | 3.10052* | Yes | **** | P<0.0001 |
|  | Good VS very good | 1.11327* | Yes | **** | P<0.0001 |

****：P<0.001,***:P<0.001,**:P<0.01;*:P<0.05

ns: no significant
