# Supplementary material for: Factors associated with academic burnout and its prevalence among university students: a cross-sectional study
Source: BMC Med Educ. 2023 May 6;23:317. doi: 10.1186/s12909-023-04316-y (PMC10163855; doi:10.1186/s12909-023-04316-y)
Supplement: Supplementary file 3 — Additional file 3. The result of pairwise comparisons among different demographic variables in reduced personal accomplishment dimension. [file 12909_2023_4316_MOESM3_ESM.docx]

**Additional file 3:The result of pairwise comparisons among different demographic variables in reduced personal accomplishment dimension**

| **Variable** |  | **Mean rank diff** | **Significant** | **Summary** | **Adjusted P Value** |
| --- | --- | --- | --- | --- | --- |
| Grade | Freshman VS. sophomore | 1.53739* | Yes | **** | P<0.0001 |
|  | Freshman VS. junior | .78686* | Yes | **** | P<0.0001 |
|  | Freshman VS. senior | 1.49091* | Yes | **** | P<0.0001 |
|  | Freshman VS. senior | 3.29553* | Yes | ** | 0.006 |
|  | Freshman VS. Master | 0.02179 | No | ns | 0.921 |
|  | Freshman VS Doctor | -0.25611 | No | ns | 0.629 |
|  | Sophomore VS. junior | -.75052* | Yes | **** | P<0.0001 |
|  | Sophomore VS. senior | -0.04648 | No | ns | 0.851 |
|  | Sophomore VS. senior | 1.75814 | No | ns | 0.141 |
|  | Sophomore VS Master | -1.51560* | Yes | **** | P<0.0001 |
|  | Sophomore VS Doctor | -1.79349* | Yes | ** | 0.001 |
|  | Junior VS. senior | .70405* | Yes | ** | 0.008 |
|  | Junior VS. Senior | 2.50866* | Yes | * | 0.036 |
|  | Junior VS Master | -.76508* | Yes | ** | 0.002 |
|  | Junior VS Doctor | -1.04297 | No | ns | 0.054 |
|  | Senior VS. Senior | 1.80462 | No | ns | 0.137 |
|  | Senior VS Master | -1.46912* | Yes | **** | P<0.0001 |
|  | Senior VS Doctor | -1.74702* | Yes | ** | 0.002 |
|  | Fifth year of college VS. Master's | -3.27374* | Yes | ** | 0.007 |
|  | Fifth year of college VS Doctor | -3.55163* | Yes | ** | 0.006 |
|  | Master VS Doctor | -0.27789 | No | ns | 0.625 |
| Whether you hold a position in the university | Student leaders (class/ student/society, etc.) VS No job | 1.34017* | Yes | **** | P<0.0001 |
|  | Student leader (class/Student union/club, etc.) VS Student leader in the past | .84386* | Yes | **** | P<0.0001 |
|  | No job VS. used to be a student leader in college | -.49631* | Yes | * | 0.011 |
| The highest level of education received by his or her parents | Junior high school and below VS junior high school | -1.17836* | Yes | **** | P<0.0001 |
|  | Junior high school and below VS High school or technical secondary school school | -1.65241* | Yes | **** | P<0.0001 |
|  | Junior high VS junior high | -1.80962* | Yes | **** | P<0.0001 |
|  | Junior high VS Undergraduate | -2.35511* | Yes | **** | P<0.0001 |
|  | Junior high school VS Master's and above | -2.68467* | Yes | **** | P<0.0001 |
|  | Junior high school VS. high school or technical school | -.47405* | Yes | **** | P<0.0001 |
|  | Junior high school VS junior college | -.63126* | Yes | **** | P<0.0001 |
|  | Junior high school VS Undergraduate | -1.17675* | Yes | **** | P<0.0001 |
|  | Junior high school VS Master's degree or above | -1.50631* | Yes | **** | P<0.0001 |
|  | High school or technical secondary school VS. junior college | -0.15721 | No | ns | 0.299 |
|  | High school or technical secondary school school VS Undergraduate | -.70270* | Yes | **** | P<0.0001 |
|  | High school or technical secondary school school VS Master's degree or above | -1.03227* | Yes | ** | 0.001 |
|  | Junior college VS Undergraduate | -.54549* | Yes | ** | 0.001 |
|  | Junior college VS Master degree or above | -.87505* | Yes | ** | 0.007 |
|  | Undergraduate VS Master's or above | -0.32956 | No | ns | 0.297 |
| Monthly living expenses(Yuan) | <1,000 VS1,000-1,500 | 0.25123 | No | ns | 0.178 |
|  | <1,000 VS1,500-2,000 | -0.00773 | No | ns | 0.967 |
|  | <1,000 VS2,500-3,000 | -.50568* | Yes | * | 0.019 |
|  | <1,000 VS>3,000 | -0.3227 | No | ns | 0.228 |
|  | 1,000-1,500 VS 1,500-2,000 | -.25896* | Yes | ** | 0.008 |
|  | 1,000-1,500 VS 2,500-3,000 | -.75691* | Yes | **** | P<0.0001 |
|  | 1,000-1,500 VS>3,000 | -.57393* | Yes | ** | 0.007 |
|  | 1,500-2,000 VS 2,500-3,000 | -.49795* | Yes | ** | 0.001 |
|  | 1,500-2,000 VS>3,000 | -0.31497 | No | ns | 0.144 |
|  | 2,500-3,000 VS>3,000 | 0.18298 | No | ns | 0.444 |
| The pressure of study and life in the past two months | 1-Extremely little stressed VS2-Very little stressed | 3.34937* | Yes | **** | P<0.0001 |
|  | 1-Extremely little stressed VS3-Little stressed | 4.88641* | Yes | **** | P<0.0001 |
|  | 1-Extremely little stressed VS4-Much stressed | 5.53415* | Yes | **** | P<0.0001 |
|  | The pressure is minimal VS5-Very much stressed | 5.97922* | Yes | **** | P<0.0001 |
|  | 1-Extremely little stressed VS 6-Extremely much stressed | 7.29555* | Yes | **** | P<0.0001 |
|  | 2-Very little stressedVS3-Little stressed | 1.53704* | Yes | **** | P<0.0001 |
|  | 2-Very little stressedVS4-Much stressed | 2.18478* | Yes | **** | P<0.0001 |
|  | 2-Very little stressedVS5-Very much stressed | 2.62985* | Yes | **** | P<0.0001 |
|  | 2-Very little stressed VS 6-Extremely much stressed | 3.94618* | Yes | **** | P<0.0001 |
|  | 3-Little stressedVS4-Much stressed | .64774* | Yes | **** | P<0.0001 |
|  | 3-Little stressedVS5-Very much stressed | 1.09281* | Yes | **** | P<0.0001 |
|  | 3-Little stressed VS 6-Extremely much stressed | 2.40914* | Yes | **** | P<0.0001 |
|  | 4-Much stressedVS5-Very much stressed | .44508* | Yes | * | 0.015 |
|  | 4-Much stressed VS6-Extremely much stressed | 1.76140* | Yes | **** | P<0.0001 |
|  | 5-Very much stressed VS 6-Extremely much stressed | 1.31633* | Yes | **** | P<0.0001 |
| The degree of interest in professional knowledge | Very interested VS Have interested | 4.24335* | Yes | **** | P<0.0001 |
|  | Very interested VS. Generally | 7.22852* | Yes | **** | P<0.0001 |
|  | Very interested VS Less interest | 6.47356* | Yes | **** | P<0.0001 |
|  | Very interested VS No interest | 10.18888* | Yes | **** | P<0.0001 |
|  | Have interested VS. Generally | 2.98517* | Yes | **** | P<0.0001 |
|  | Have interested VS are Less interest | 2.23021* | Yes | **** | P<0.0001 |
|  | Have interested VS. No interest | 5.94553* | Yes | **** | P<0.0001 |
|  | General VS is Less interest | -.75496* | Yes | **** | P<0.0001 |
|  | Generally VS No interest | 2.96036* | Yes | **** | P<0.0001 |
|  | Less interest VS No interest | 3.71532* | Yes | **** | P<0.0001 |
| Weekly exercise time (hours) | 1 hour VS2 hours | -1.78977* | Yes | **** | P<0.0001 |
|  | 1 hour VS3 hours | -3.21258* | Yes | **** | P<0.0001 |
|  | 1 hour VS4 hours | -4.53581* | Yes | **** | P<0.0001 |
|  | 2 hours VS3 hours | -1.42280* | Yes | **** | P<0.0001 |
|  | 2 hours VS4 hours | -2.74604* | Yes | **** | P<0.0001 |
|  | 3 hours VS4 hours | -1.32323* | Yes | **** | P<0.0001 |
| Smoking | Smoking VS Quit smoking | -0.0808 | No | ns | 0.816 |
|  | Smoking VS Never smoked | -0.22776 | No | ns | 0.287 |
|  | Quit smoking VS Never smoked | -0.14696 | No | ns | 0.601 |
| Drinking | Drinking VS Quit Drinking | -.53079* | Yes | * | 0.019 |
|  | Drinking VS Never drank | -1.20790* | Yes | **** | P<0.0001 |
|  | Quit drinking VS Never drank | -.67710* | Yes | ** | 0.002 |
| Overall satisfaction with study | Very satisfied VS Satisfied | 4.96911* | Yes | **** | P<0.0001 |
|  | Very satisfied VS Generally | 8.55664* | Yes | **** | P<0.0001 |
|  | Very satisfied VS Dissatisfied | 10.78364* | Yes | **** | P<0.0001 |
|  | Very satisfied VS Very dissatisfied | 12.85457* | Yes | **** | P<0.0001 |
|  | Satisfied VS Generally | 3.58753* | Yes | **** | P<0.0001 |
|  | Satisfied VS Dissatisfied | 5.81453* | Yes | **** | P<0.0001 |
|  | Satisfied VS very Very dissatisfied | 7.88546* | Yes | **** | P<0.0001 |
|  | Generally VS Dissatisfied | 2.22700* | Yes | **** | P<0.0001 |
|  | Generally VS Very dissatisfied | 4.29793* | Yes | **** | P<0.0001 |
|  | Dissatisfied VS Very dissatisfied | 2.07093* | Yes | **** | P<0.0001 |
| Sleep quality in the past two months | Very bad VS pretty bad | -.98004* | Yes | ** | 0.004 |
|  | Very poor VS average | -2.05270* | Yes | **** | P<0.0001 |
|  | Very bad VS good | -4.00311* | Yes | **** | P<0.0001 |
|  | Very bad VS very good | -6.75574* | Yes | **** | P<0.0001 |
|  | Pretty bad VS so-so | -1.07267* | Yes | **** | P<0.0001 |
|  | Pretty bad VS pretty good | -3.02307* | Yes | **** | P<0.0001 |
|  | Pretty bad VS very good | -5.77570* | Yes | **** | P<0.0001 |
|  | VS in general is fine | -1.95040* | Yes | **** | P<0.0001 |
|  | VS in general is very good | -4.70303* | Yes | **** | P<0.0001 |
|  | Good VS very good | -2.75263* | Yes | **** | P<0.0001 |

****：P<0.001,***:P<0.001,**:P<0.01;*:P<0.05

ns: no significant
